# Exploring phylosymbiosis in the skin microbiome of coral reef fish: synergistic effects of environmental and host-specific factors

**DOI:** 10.1093/ismeco/ycag121

**Published:** 2026-05-07

**Authors:** Han Lai, Kuo Gao, Lan Qiu, Mingpan Huang, Wen Yu, Hao Li, Zhaojie Peng, Shichao Wei, Fuwen Wei, Wenliang Zhou

**Affiliations:** Center for Evolution and Conservation Biology, Southern Marine Science and Engineering Guangdong Laboratory (Guangzhou), Guangzhou 511458, China; Laoshan Laboratory Guangdong Institute, Guangzhou 511458, China; Center for Evolution and Conservation Biology, Southern Marine Science and Engineering Guangdong Laboratory (Guangzhou), Guangzhou 511458, China; Center for Evolution and Conservation Biology, Southern Marine Science and Engineering Guangdong Laboratory (Guangzhou), Guangzhou 511458, China; Department of Ocean Science and Otto Poon Center for Climate Resilience and Sustainability, The Hong Kong University of Science and Technology, Hong Kong, China; Center for Evolution and Conservation Biology, Southern Marine Science and Engineering Guangdong Laboratory (Guangzhou), Guangzhou 511458, China; Center for Evolution and Conservation Biology, Southern Marine Science and Engineering Guangdong Laboratory (Guangzhou), Guangzhou 511458, China; Center for Evolution and Conservation Biology, Southern Marine Science and Engineering Guangdong Laboratory (Guangzhou), Guangzhou 511458, China; Department of Ocean Science and Otto Poon Center for Climate Resilience and Sustainability, The Hong Kong University of Science and Technology, Hong Kong, China; Center for Evolution and Conservation Biology, Southern Marine Science and Engineering Guangdong Laboratory (Guangzhou), Guangzhou 511458, China; Center for Evolution and Conservation Biology, Southern Marine Science and Engineering Guangdong Laboratory (Guangzhou), Guangzhou 511458, China; Laoshan Laboratory Guangdong Institute, Guangzhou 511458, China; Center for Evolution and Conservation Biology, Southern Marine Science and Engineering Guangdong Laboratory (Guangzhou), Guangzhou 511458, China; College of Forestry, Jiangxi Agricultural University, Nanchang 330045, China; Key Laboratory of Animal Ecology and Conservation Biology, Institute of Zoology, Chinese Academy of Sciences, Beijing 100101, China; Center for Evolution and Conservation Biology, Southern Marine Science and Engineering Guangdong Laboratory (Guangzhou), Guangzhou 511458, China; Laoshan Laboratory Guangdong Institute, Guangzhou 511458, China

**Keywords:** coral reef fish, skin microbiome, environmental filtering, *16S rRNA* gene, Phylosymbiosis, South China Sea

## Abstract

The skin is the largest organ in vertebrates, and its microbiome plays a crucial role in immune function and host health. Coral reef fish, characterized by rapid adaptive radiation and complex functional diversity, provide an ideal model for studying the assembly of vertebrate skin microbiomes and the mechanisms that drive this process. In this study, we used *16S rRNA* gene amplicon sequencing to investigate the factors influencing the skin microbiomes of coral reef fish across a broad spatial scale. We analyzed 447 skin microbiome samples from 138 fish species, collected from different regions along the latitudinal gradient in coral reef ecosystems of the South China Sea. Our results revealed significant differences in the skin microbiomes of coral reef fish between coastal and offshore habitats in terms of taxonomic composition, diversity, and predicted functional potential. The microbiome structure was influenced by a combination of environmental factors, host traits, and phylogenetic relationships. Notably, we observed a phylosymbiotic pattern in the skin microbiomes of coral reef fish, with more closely related fish exhibiting more similar microbial communities. Temperature and silicate concentration were identified as the primary environmental drivers of microbial community structure, while host-specific factors such as mobility and diet indirectly influenced microbiome assembly by shaping host-associated microenvironment and feeding habitats. Our study highlights the complex interplay between environmental and host-specific factors in shaping coral reef fish skin microbiomes and provides new insights into host–microbe interactions in coral reef ecosystems.

## Introduction

Microorganisms form specific symbiotic relationships with their hosts and play essential roles in critical biological processes, such as energy metabolism, nutrient cycling, and immune defense [1–4]. The skin is the largest organ in fish, and its mucus is a crucial part of the immune system, providing a barrier against pathogens [5, 6]. A diverse and stable skin microbiome is essential for preserving the integrity of this defense system [5]. Some skin-associated microbes produce antimicrobial compounds that help fish combat pathogenic microorganisms, thereby promoting host health and resilience [7, 8]. Additionally, interactions between the skin microbiota and host mucosal immunity contribute to barrier stability by modulating immune responses and maintaining mucosal homeostasis [9, 10]. Consequently, the health of fish skin microbiomes serves as a direct indicator of the health of both the fish and their surrounding ecosystems.

The assembly of fish skin microbiomes is shaped by the interaction among biotic factors (e.g. host-specific factors) and abiotic environmental variables [7, 11]. While the immediate ocean environment is the primary source of microbial communities on fish skin, numerous studies have shown that the microbial composition of marine fish skin differs significantly from that of the surrounding seawater [11, 12]. Phylosymbiosis, a widely recognized phenomenon across animal taxa, refers to a pattern in which the similarity of host-associated microbial communities covaries with host phylogenetic relatedness [13, 14], such that more closely related hosts tend to harbor more similar microbiomes [15]. This pattern suggests that microbial colonization on fish skin may involve host-specific selective filtering mechanisms [11, 16]. In addition to host-specific filtering, selection imposed by external abiotic environmental conditions also plays a crucial role in the assembly and evolutionary adaptation of fish skin microbiomes [11, 17, 18]. These selection processes highlight the interplay of environmental filtering and host evolution in determining unique host-microbiome interactions [11, 19]. However, the extent to which host-specific factors contribute to the assembly of fish skin microbiomes, and how host-specific and environmental factors jointly shape the composition of fish skin microbial communities, remain poorly understood [11].

Coral reef ecosystems, often referred to as the “tropical rainforests of the ocean” due to their immense biodiversity and ecological complexity, support ~30% of marine species [20–22]. Coral reef fish, which have evolved through rapid evolutionary radiations [23], are essential components of coral reef ecosystems, playing key roles in maintaining ecosystem functionality through energy and material transfer involving diverse trophic and symbiotic interactions [24, 25]. However, due to anthropogenic activities, environmental pollution, and frequent marine heatwaves, serious degradation of coral reef ecosystems is accelerating worldwide, potentially affecting the skin microbial homeostasis and health of coral reef fish [26, 27]. Understanding the assembly of skin microbial communities in coral reef fish, and how these communities adapt and respond to environmental changes, is crucial for the conservation of both coral reef fish and the broader coral reef ecosystems.

Coral reef fish harbor a rich diversity of commensal microorganisms and are considered reservoirs and carriers of microbial communities [24]. The typical characteristics of coral reef fish and their reef environment, including rapid evolution associated with the phenotypic plasticity of adaptive traits and dynamic habitat conditions, provide an ideal model for investigating the primary factors shaping animal microbiome structure [28]. However, studies examining the effects of phylosymbiosis and environmental heterogeneity on the selective colonization and assembly of skin microbiomes in coral reef fish remain largely limited, with existing studies focusing primarily on small-scale regions and a limited number of taxa [12]. The coral reef ecosystems of the South China Sea, characterized by their high diversity of reef-associated fish and spanning a broad latitudinal gradient, include both coastal and offshore reefs subjected to varying levels of environmental disturbance [29, 30]. This makes the region an ideal natural laboratory for studying the diversity of fish skin microbiomes and the mechanisms underlying their assembly.

In this study, we used large-scale sampling of coral reef fish and amplicon sequencing of their skin microbiomes, combined with bioinformatics analyses, phylogenetic tree construction, and multivariate statistical analyses, to examine the phylosymbiotic patterns of coral reef fish skin microbiomes and to identify the key factors shaping these microbial communities. We focused on two primary drivers of microbial diversity: (i) environmental variables (e.g. temperature, pH, nutrients) and habitat type (coastal *vs.* offshore); and (ii) host-specific factors, including taxon relatedness, traits (e.g. size, mobility, activity period, and diet), and phylogeny. We hypothesized that fish skin microbiome assembly is jointly structured by environmental and host-specific factors. Specifically, we predicted that habitat type and key environmental gradients would primarily account for spatial variation, whereas host traits and phylogenetic relatedness would explain interspecific differences and contribute to a phylosymbiotic pattern. Our findings are crucial for developing conservation strategies that address both macro- and microbial biodiversity, thereby enhancing our understanding of the resilience of coral reef ecosystems to anthropogenic pressures.

## Materials and methods

### Study area and sample collection

**Figure 1:**
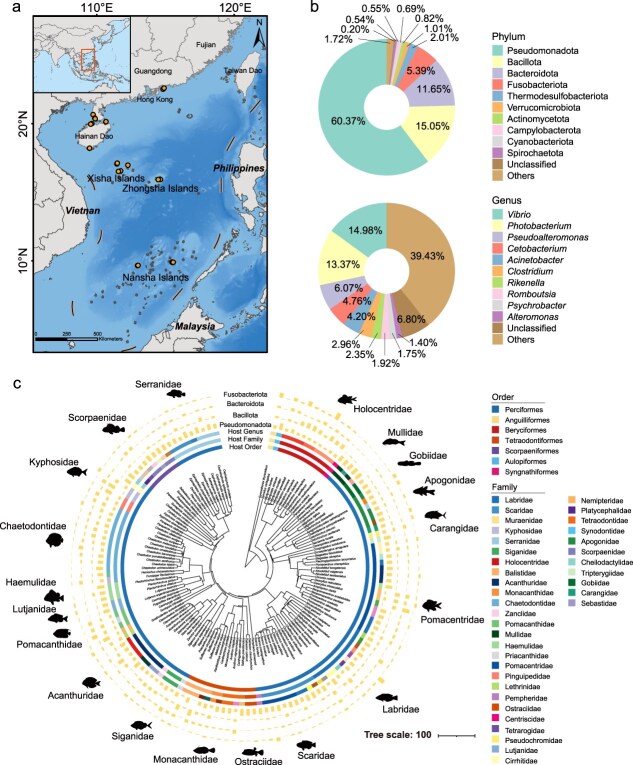
Overview of the coral reef fish skin microbiome in the South China Sea. (a) Spatial distribution of the sampling sites. (b) Pie chart depicting the relative abundance of bacterial phyla and genera in the skin microbiomes of all fish samples. (c) A phylogenetic tree based on data from 138 coral reef fish species, illustrating the phylum-level composition of the skin microbiome along with host taxonomic attributes. The concentric rings, moving outward, display the host taxonomy (order, family, genus) in succession. The outermost bar chart shows differences in the composition of dominant bacterial phyla, including Pseudomonadota, Bacillota, Bacteroidota, and Fusobacteriota, across different fish families. Silhouette images of representative fish species are provided for context. The host genus color legend is omitted for clarity.

Fish skin microbiome samples were collected during March to October of both 2022 and 2023 at 25 sites across the South China Sea. The study area spans latitudes from 9.6°N to 22.6°N, covering a range of 13 degrees of latitude and including both coastal and offshore coral reef ecosystems, each subject to varying degrees of environmental disturbance. Coastal coral reefs (near Guangdong Province and Hainan Province) are located at higher latitudes and are more affected by anthropogenic activities than offshore coral reefs (around Xisha, Zhongsha, and Nansha Islands), which are situated at lower latitudes. Fish specimens were captured by hand during dives and using gill nets at depths of <18 m, and species were primarily identified based on morphological characteristics, with molecular barcoding used when morphology was inconclusive. Skin microorganisms were sampled by swabbing a standardized area on the lateral flank of each individual using sterile swabs (Tenke). Swabs were placed into sterile 2 ml microcentrifuge tubes without preservation buffer and immediately stored at −40°C in the field, and subsequently transferred to −80°C upon arrival at the laboratory for long-term storage prior to DNA extraction. A total of 447 skin microbiome samples were collected from 138 fish species representing 80 genera, 37 families, and 7 orders (Fig. 1a and Supplementary Data 1). The number of individuals sampled per species ranged from 1 to 25 (median = 2 individuals per species). Of these, 134 samples from 52 species were obtained from coastal coral reef fish, and 313 samples from 88 species were obtained from offshore coral reef fish.

Environmental parameters (Supplementary Data 2), including seawater temperature (Temp), salinity, pH, dissolved oxygen (DO), nitrate concentration (NO₃), dissolved inorganic carbon (DIC), phosphate concentration (PO₄), silicate concentration (SI), chlorophyll-a levels (Chla), seawater velocity (SWV), and net primary production, were obtained for each sampling site on the corresponding sampling date from the Copernicus Marine MyOcean Viewer (https://data.marine.copernicus.eu/viewer), with a spatial resolution of 1/12°. Environmental variables with a Variance Inflation Factor below 5 were selected based on collinearity analysis to ensure statistical independence. The selected environmental variables were used in subsequent analyses of environmental impacts on microbial diversity and functionality.

### DNA extraction and *16S rRNA* gene sequencing

Skin swabs were placed in 2 ml centrifuge tubes, 600 μl of lysis buffer and 15 μl of Proteinase K were added and incubated at 70°C for 15 min (TIANamp Stool DNA Kit). Afterwards, DNA was extracted according to the manufacturer’s protocol. Primers 338F (5′-ACTCCTACGGGGAGGCAGCA-3′) and 806R (5′-GGACTACHVGGGTWTCTAAT-3′) [31] were used for PCR amplification of the highly variable V3-V4 region of the *16S rRNA* gene, with each sample amplified in triplicate and the resulting products subsequently pooled. The PCR reaction was conducted on a prepared sample comprising 10 ng of template DNA, 0.8 μl of each forward and reverse primer, 10 μl of 2× Pro Taq Master Mix, and ddH_2_O, made up to 20 μl. The amplification procedure was as follows: 95°C for 3 min, followed by 30 cycles of 95°C for 30 s, annealing at 53°C for 30 s, and extension at 72°C for 45 s. After the final cycle, the reaction was held at 72°C for 10 min and then stored at 10°C until subsequent analyses. PCR blanks were included in each sequencing run to monitor contamination, and no amplicons or reads were detected.

Pooled PCR products were detected by gel electrophoresis using 2% agarose. The products were recovered and purified using the AxyPrep DNA Gel Extraction Kit (Axygen Biosciences, Union City, CA, USA) and the recovered products were quantified using Quantus™ Fluorometer (Promega, USA). Sequencing was performed on an Illumina NextSeq 2000 platform using PE300 paired-end sequencing.

### Sequence processing and taxonomic assignment

Raw *16S rRNA* gene amplicon sequencing data were processed using QIIME2 (version 2025.4.0) [32]. Initially, adapter and primer sequences were removed using the Cutadapt plugin. Quality control was performed with the DADA2 plugin, which included merging paired-end reads, filtering out low-quality sequences, and denoising to infer amplicon sequence variants (ASVs; Supplementary Data 3). All steps followed the standard QIIME2 workflow to ensure data accuracy and quality. Taxonomic assignment of ASVs was performed using the SILVA v.138.2 database. Non-target sequences, including mitochondrial and chloroplast sequences, were excluded, and ASVs with fewer than 10 total reads across all samples were removed. The resulting ASV table was rarefied to 15 142 reads per sample (*n* = 447) for alpha and beta diversity analyses (Supplementary Data 4). Summary statistics of reads retained across all processing steps are provided in Supplementary Table 1.

### Host phylogeny and traits

The phylogenetic tree of fish species was constructed using the *rtrees* package in R [33], which generates phylogenies by matching species to the Fish Tree of Life megatree [34] and grafting unmatched taxa at the most taxonomically appropriate level. The constructed tree was visualized and annotated in detail using iTOL v7 [35]. The concentric rings of the phylogenetic tree represent taxonomic hierarchies (order, family, genus), while the outermost bar chart illustrates the relative abundance of major bacterial phyla. Fish silhouettes were obtained from the R package *fishualize* (v0.2.3) [36]. Additionally, the constructed phylogenetic tree was further used to calculate phylogenetic distances between host species and applied in subsequent Mantel tests and local indicator of phylogenetic association (LIPA) analyses.

The ecology of the 138 species was described using four traits [12], including maximum body size, mobility, activity period, and diet, obtained from FishBase (http://www.fishbase.org) and the global database of functional traits for reef fishes as reported in relevant literature [37–39] (Supplementary Table 2 and Supplementary Data 1). To assess whether these traits were phylogenetically conserved, we quantified phylogenetic signal at the species level using Blomberg’s K and Pagel’s λ for body size, and Fritz and Purvis’ D statistic for categorical traits, with significance evaluated using 9999 permutations [40, 41].

### Relative contributions of environmental and host-specific factors to microbial community assemblages

To assess the relative contributions of environmental factors, host traits, host phylogeny, and geographic distance to microbial community dissimilarity, we performed a Multiple Regression on distance Matrices (MRM) using the *ecodist* R package [42]. Distance matrices were constructed for the microbial data (using Bray–Curtis dissimilarity), environmental variables (using Euclidean distance), host traits (using Gower distance for categorical and ordinal variables), and geographic distance (great-circle distance calculated from latitude and longitude coordinates). Host phylogenetic distance was quantified using the *cophenetic* function, based on the branch length distances (patristic distances) derived from the phylogenetic tree.

Using the MRM model, we analyzed the relationships between microbial community structure (represented by Bray–Curtis dissimilarity) and environmental as well as host-specific predictors. Geographic distance was included to account for potential spatial autocorrelation among samples. We examined the overall and specific influences of environmental variables, host traits, phylogenetic and geographic distance on microbial community structure. Each model was evaluated using 9999 permutations to assess the statistical significance of each predictor. The magnitude of the regression coefficients reflects the influence of each variable on microbial community structure, while the *R^2^* value indicates the proportion of variance explained by the fitted model.

### Influence of host-specific factors on fish skin microbiome

To evaluate the influence of host traits on fish skin microbiome structure, Bray–Curtis dissimilarities were analyzed using PERMANOVA (*adonis2* in the *vegan* package; 999 permutations) for each trait category, followed by pairwise PERMANOVA comparisons with multiple-testing correction (*pairwiseAdonis*). To explore the relationship between microbial community β-diversity and host phylogenetic distances, we conducted a Mantel test using the *mantel* function in the *vegan* R package. Microbial β-diversity was quantified using Bray–Curtis dissimilarity matrices (*vegan* package), while host phylogenetic distances were represented by cophenetic distance matrices derived from the constructed phylogenetic tree (*ape* package). The Mantel test used Spearman’s rank correlation to assess the association between the two matrices, with statistical significance determined by 999 permutations. Additionally, we performed linear regression analysis to further investigate the relationship between Bray–Curtis dissimilarity and host phylogenetic distances, visualizing the trends and evaluating the strength of their linear association.

LIPA analysis was conducted to explore phylogenetic signals within microbial community assemblages. Phylogenetic signals of ASVs and LIPA (local Moran’s I) were calculated using the *phylosignal* R package [43], with significance assessed through 9999 permutations. LIPA values greater than 0 were retained and visualized as a heatmap on the phylogenetic tree.

### Statistical analyses of microbial diversity

*Alpha* diversity indices, including Shannon, Simpson, Pielou’s evenness, and richness, were calculated using the vegan package in R, with group differences evaluated by Wilcoxon rank-sum tests. Canonical Correspondence Analysis (CCA) was performed to explore the influence of environmental factors on the microbial community structure of coral reef fish skin, while Permutational Multivariate Analysis of Variance (PERMANOVA) analysis (*adonis2* function) was used to test the significance of differences in community structure between coastal and offshore coral reef fish. Linear Discriminant Analysis Effect Size (LEfSe) was used to identify bacterial taxa that significantly differed in abundance between coastal and offshore regions, using an LDA score threshold of >4.0.

### Functional analysis of microbial communities

The non-rarefied ASV table obtained from QIIME2 was converted into PICRUSt2 format, and *16S rRNA* gene copy numbers were predicted using PICRUSt2 (v2.5.2) [44]. The corresponding KEGG Ortholog (KO) identifiers were then inferred. Based on the identified KO identifiers, the corresponding level 1 and 2 metabolic pathways were retrieved from the KEGG database. KEGG pathway enrichment analysis was performed to identify the top 10 enriched level 3 metabolic pathways in the skin microbiota of fish from both coastal and offshore regions. Additionally, LEfSe analysis (LDA score > 2.8) was performed to identify significantly different pathways in level 3 metabolic pathways between the coastal and offshore regions. Finally, CCA analysis was performed to examine the relationship between environmental factors and the functional structure of microbial communities. PERMANOVA was then used to test the significance of differences in microbial community functional structure between coastal and offshore coral reef fish.

## Results

### Fish skin microbiome composition from the South China Sea

Fish skin microbiome samples (*n* = 447) were collected from coral reef ecosystems in the South China Sea, spanning the latitudinal gradient from 9.6°N to 22.6°N (Fig. 1a). These samples represent 138 fish species across 7 orders, 37 families, and 80 genera. Rarefaction analyses confirmed that sampling depth sufficiently sampled skin microbiome diversity (Supplementary Fig. 1). At the phylum level, the skin microbiome of coral reef fish in the South China Sea predominantly comprised Pseudomonadota, Bacillota, Bacteroidota, and Fusobacteriota, which together accounted for ~92.46% of the community (Fig. 1b). The most abundant genera included *Vibrio* (14.98%), *Photobacterium* (13.37%), *Pseudoalteromonas* (6.07%), *Cetobacterium* (4.76%), and *Acinetobacter* (4.20%).

The host phylogenetic tree annotated with skin microbiome composition shows that Pseudomonadota is the most common phylum (Fig. 1c), being detected in all 447 fish samples from 138 species, and dominated the skin microbiome of most fish species. However, the relative abundances of Bacillota, Bacteroidota, and Fusobacteriota varied considerably among fish species, likely reflecting host-specific factors or the influence of environmental factors on skin microbial community composition.

### Relative contribution of environmental and host-specific factors to the skin microbiome

**Figure 2:**
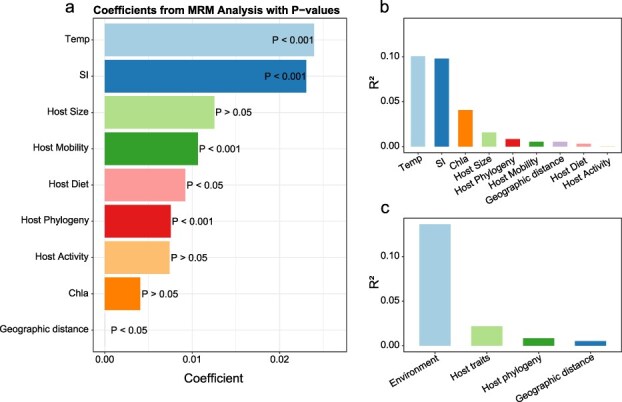
Relative contribution of environmental and host-specific factors to the skin microbiome from multiple regression on distance matrices (MRM) analysis. (a) Regression coefficients for individual environmental, host trait, phylogenetic, and geographic distance predictors, indicating the specific influence of each factor on skin microbiome dissimilarity. (b) Proportion of variance accounted for by individual environmental, host traits, phylogenetic and geographic variables, representing their relative contributions to variation in skin microbiome dissimilarity. (c) Summary of the comparative variance accounted for by environmental, host traits, phylogenetic and geographic factors. Note: Temp refers to temperature, SI refers to silicate concentration, and Chla refers to chlorophyll-a.

MRM analysis of the relative contributions of environmental and host-related factors to the composition of coral reef fish skin microbiomes (Fig. 2) indicates that environmental factors accounted for the largest proportion of explained variance (*R*^2^ = 0.136), substantially exceeding the explanatory power of host traits (*R*^2^ = 0.022), host phylogeny (*R*^2^ = 0.008), and geographic distance (*R*^2^ = 0.005). Specifically, temperature (coefficient = 0.024, *R*^2^ = 0.101, *P* < .001) and silicate concentration (coefficient = 0.023, *R*^2^ = 0.098, *P* < .001) most strongly influenced microbial community structure. These findings emphasize the critical role of environmental conditions in shaping the composition of coral reef fish skin microbiomes. Among host-specific factors, mobility and diet were significantly associated with skin microbial community dissimilarity (*P* < .001 and *P* < .05, respectively), while host size and activity period showed no significant association (*P* > .05). Host phylogeny also displayed a significant correlation with skin microbial community dissimilarity (*P* < .001). However, these putative evolutionary relationships among host species exerted much less influence than the immediate environmental factors and functional traits of the hosts (Fig. 2).

### Influence of host-specific factors on fish skin microbiome

**Figure 3:**
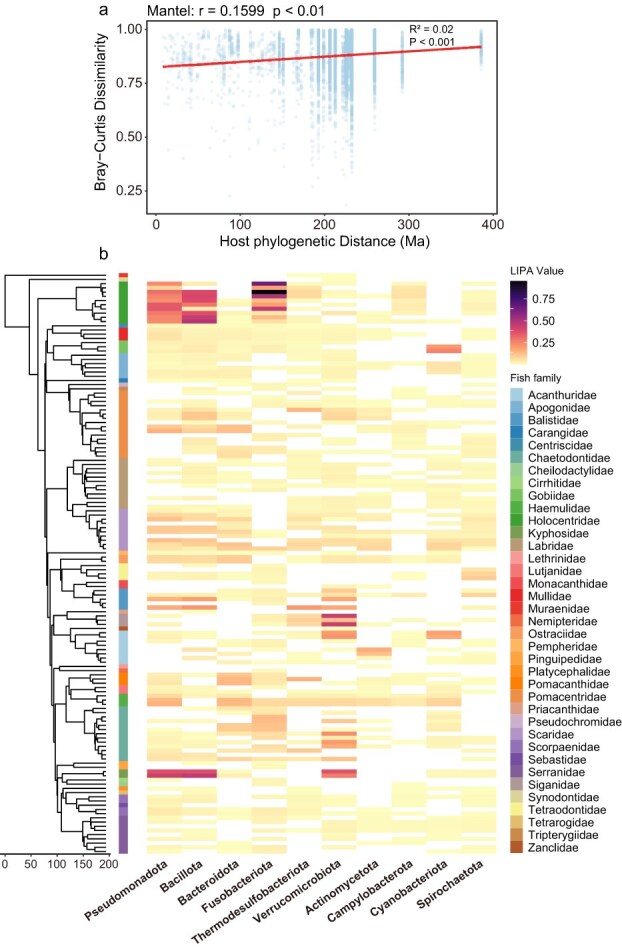
Phylosymbiosis in coral reef fish skin microbiota: (a) relationship between Bray-Curtis dissimilarity of skin microbiomes and host phylogenetic distance (Ma). (b) Phylogenetic signals revealed by local indicator of phylogenetic association (LIPA) analysis.

Although environmental factors predominantly shape the overall assembly of skin microbial communities, host-specific factors were also important in shaping the fine-scale structure of the skin microbiome. PERMANOVA analyses revealed significant differences in Bray–Curtis dissimilarities among categories of host size, mobility, activity period, and diet (Supplementary Fig. 2; Supplementary Table 3). In addition, microbial community dissimilarity was significantly correlated with host phylogenetic distance (Mantel *r* = 0.1599, *P* < .01) (Fig. 3a). Thus, the presence of a weak phylogenetic signal in the microbiome composition indicates that host phylogenetic distance partially influences the community structure of coral reef fish skin microbiomes. Host traits also exhibited significant phylogenetic signal. Body size showed clear phylogenetic conservatism (Blomberg’s *K* = 0.535, *P* = 1.0 × 10^−4^; Pagel’s λ = 0.889, *P* = 2.44 × 10^−14^), and most mobility, activity, and diet categories deviated significantly from random expectation based on Fritz and Purvis’ D statistic (*P* < .01; Supplementary Tables 4 and 5), with several categories showing strong phylogenetic clustering (D < 0).

We investigated the phylogenetic distribution of skin microbiome assemblages across various coral reef fish hosts using LIPA analysis (Fig. 3b). The LIPA heatmap reflects the degree of phylogenetic conservatism associated with host evolutionary relationships. Overall, most microbial phyla exhibit relatively low LIPA values, suggesting weak phylogenetic conservatism in the skin microbiomes of coral reef fish. However, certain phyla, such as Pseudomonadota, Bacillota, and Fusobacteriota, display notably larger LIPA values (indicated by dark purple regions), particularly in specific fish families such as Holocentridae (order Beryciformes) and Kyphosidae (order Perciformes), which show significant phylogenetic signals. These findings reveal variation in phylogenetic conservatism within the coral reef fish skin microbiome community, suggesting that although the overall phylosymbiotic signal is weak, specific host phylogenetic relationships still play a crucial role in shaping microbial community structure.

### Habitat and environmental correlates of fish skin microbiomes

Given the dominant role of environmental factors in shaping microbial communities, we further investigated the structural and functional characteristics of fish skin microbiomes in coastal versus offshore regions, which differ significantly in environmental conditions and the intensity of anthropogenic disturbances. Environmental factor analysis revealed significant differences between coastal and offshore regions in various environmental parameters (such as temperature, pH, chlorophyll-a, silicate, and others), with the exception of salinity (Supplementary Fig. 3). Specifically, the coastal region exhibited significantly higher levels of pH, silicate concentration, nitrate concentration, dissolved oxygen, net primary production, and chlorophyll-a compared to the offshore region, while temperature, seawater velocity, dissolved inorganic carbon, and phosphate concentration were significantly higher in the offshore region than in the coastal region.

**Figure 4:**
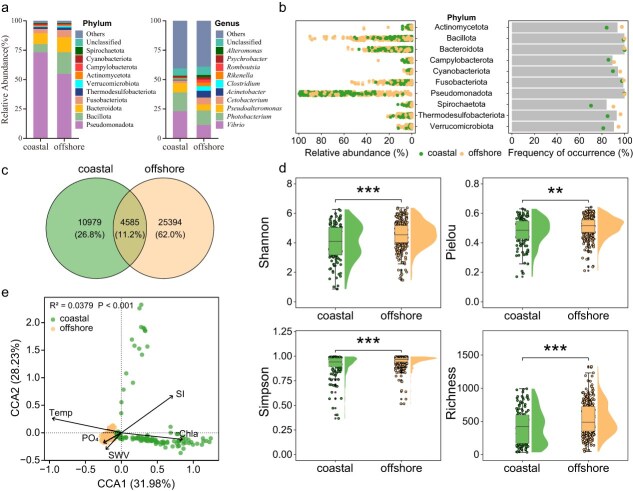
Habitat-related variation in coral reef fish skin microbiome composition and diversity: (a) relative abundance of the top 10 skin bacterial taxa at the phylum and genus levels in coastal and offshore regions; (b) dot plot showing the overall distribution of relative abundance (left) and frequency of occurrence (right) of microbial taxa in total fish (bar) and coastal or offshore coral fish (dot) at the bacterial phylum level; (c) Venn diagram illustrating shared and unique ASVs between fish skin microbiota from different habitats; (d) *Alpha* diversity indices for fish skin microbiota in coastal and offshore regions; (e) *Beta* diversity analysis of fish skin microbiota using CCA, illustrating the influence of environmental factors on fish microbial community structure. Temp refers to temperature, SI refers to silicate concentration, Chla refers to chlorophyll-a, PO₄ refers to phosphate concentration, and SWV refers to seawater velocity.

There was a significant influence of habitat on the composition of the coral reef fish skin microbiome. Among the 447 samples collected from both coastal and offshore reef fish, a total of 40 958 ASVs were identified by *16S rRNA* gene sequencing, of which 4585 (11.2%) were shared between coastal and offshore habitats (Fig. 4c). Pseudomonadota dominated the skin microbiomes of both coastal and offshore coral reef fish at the phylum level, but its relative abundance was higher in coastal regions (73.29%) than in offshore regions (54.85%) (Fig. 4a). Conversely, Bacillota exhibited a lower relative abundance in coastal regions (6.92%) compared to offshore regions (18.53%). At the genus level, *Vibrio* showed a higher relative abundance in coastal regions (23.17%) than in offshore regions (11.47%). Pseudomonadota and Bacteroidota were abundantly present at nearly all sites in both habitats; in contrast, phyla such as Thermodesulfobacteriota, Verrucomicrobiota, Actinomycetota, and Spirochaetota were more abundant at offshore sites (Fig. 4b). Through LEfSe analyses (LDA score > 4), we identified representative and significantly distinct fish skin microbial taxa between coastal and offshore coral reef habitats (Supplementary Fig. 4). Species such as *Vibrio fortis*, *Vibrio alfacsensis*, *Aliivibrio fischeri*, and *Cognatiyoonia sediminum* were markedly enriched in coastal coral reef fish samples, whereas *Photobacterium damselae*, *Vibrio ponticus*, and *Vibrio stylophorae* exhibited higher abundance in offshore samples. Additionally, *alpha* diversity indices, including Shannon, Simpson, and Pielou’s evenness, were significantly higher in offshore samples than in coastal samples (Fig. 4d). CCA and PERMANOVA tests confirmed significant differences in the assemblage structure of fish skin microbiomes between coastal and offshore habitats (*R^2^* = 0.0379, *P* < .001), with temperature, chlorophyll-a, and silicate concentration identified as the primary environmental factors shaping community structure (Fig. 4e).

**Figure 5:**
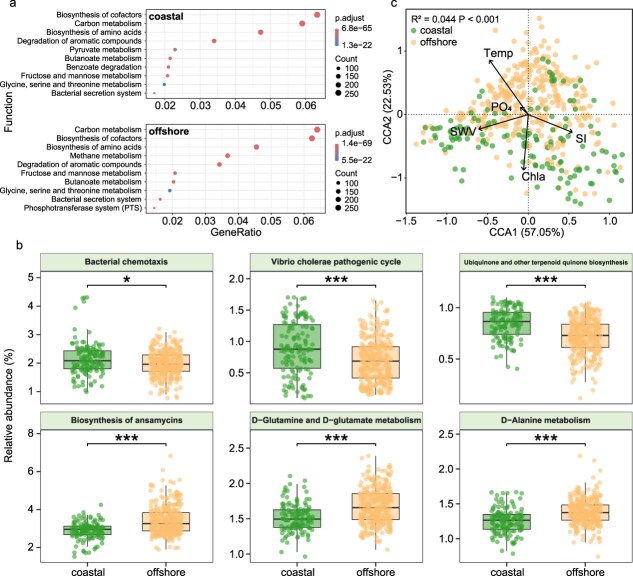
Predicted functional profiles of coral reef fish skin microbiome across habitats. (a) KEGG pathway enrichment analysis of coastal and offshore coral reef fish skin microbiomes; (b) box plots of the relative abundance of selected pathways for samples from two different habitats determined by LEfSe analysis (LDA score > 2.8). Asterisks indicate significant differences between coastal and offshore groups (^*^P < 0.05; ^**^P < 0.01; ^***^P < 0.001); (c) canonical correspondence analysis (CCA) illustrating the influence of environmental factors on microbial functional composition. Temp refers to temperature, SI refers to silicate concentration, Chla refers to chlorophyll-a, PO₄ refers to phosphate concentration, and SWV refers to seawater velocity.

To explore the influence of environmental factors on the functional diversity of microbial communities, we first examined the level 1 metabolic pathways of coral reef fish skin microbiomes between habitats (Supplementary Fig. 5). The functions of the skin microbiomes were primarily concentrated in four metabolic pathways: metabolism, genetic information processing, environmental information processing, and cellular processes, with metabolic pathways being significantly more important in offshore regions than in coastal regions. Further KEGG level 3 metabolic pathway enrichment analysis (Fig. 5a) revealed that the top 10 KEGG pathways enriched in both coastal and offshore samples were generally similar, including pathways for “Biosynthesis of cofactors,” “Carbon metabolism,” and “Biosynthesis of amino acids.” Some differences were observed between habitats, specifically the “Pyruvate metabolism” and “Benzoate degradation” pathways were more enriched in coastal microbiomes, whereas “Methane metabolism” and the “Phosphotransferase system (PTS)” were more prominent in offshore microbiomes. There were significant differences in functional pathways between habitats, with “Bacterial chemotaxis,” the “*Vibrio cholerae* pathogenic cycle,” and “Ubiquinone and other terpenoid quinone biosynthesis” significantly enhanced in coastal samples (LDA ≥ 2.8; Fig. 5b). In contrast, functional pathways such as “Biosynthesis of ansamycins,” “D-Glutamine and D-glutamate metabolism,” and “D-Alanine metabolism” were more enriched in offshore samples. These differences suggest that environmental conditions in the habitats may drive functional shifts in microbial communities, as they adapt to their respective ecological niches.

Finally, we used canonical correspondence analysis (CCA) to assess the influence of environmental factors on the functional structure of the microbial communities (Fig. 5c). Environmental factors contributed 57.05% and 22.53% to the observed variance on CCA1 and CCA2, respectively, accompanied by a significant effect on the functional structure of the skin microbiota (*R^2^* = 0.044, *P* < .001). Among these, differences in functional structure between coastal and offshore microbial communities were primarily affected by temperature and chlorophyll-a.

## Discussion

Skin microbial communities play an essential role in maintaining the health and homeostasis of coral reef fish [5]. In the present study, we demonstrate that the skin microbiomes of coral reef fish across different regions and taxa are dominated by the phylum Pseudomonadota, with prevalent genera including *Vibrio*, *Photobacterium*, and *Pseudoalteromonas*. This dominance is consistent with previous studies of marine fish skin microbiomes, highlighting the ubiquity and adaptability of these microbial taxa among marine fish [22, 45]. Although some *Vibrio* species are recognized pathogens of marine fish under certain conditions, vibrios are ubiquitous in marine environments and are commonly associated with diverse marine animals, spanning commensal, opportunistic, and pathogenic lifestyles [46]. Their enrichment in coastal habitats may reflect environmentally mediated shifts toward disturbance-tolerant or opportunistic taxa. *Pseudoalteromonas* has been widely reported as a surface-associated, biofilm-forming marine bacterium that produces extracellular inhibitory compounds [47], whereas *Photobacterium* is frequently detected in marine fish skin microbiomes and includes lineages that can behave as opportunistic or pathogenic associates [45]. Together, the ecological versatility of these dominant taxa suggests that fish skin selects for metabolically flexible and surface-adapted bacteria capable of thriving in the highly dynamic and host-influenced mucus microenvironment. The skin of marine fish serves as a critical interface for interactions with the external environment, and the stable and conserved composition of the skin microbial community highlights the important role of commensal microorganisms in establishing a protective barrier in coral reef fish.

Significant differences in the taxonomic composition and diversity of the skin microbiomes were observed between coral reef fish from coastal and offshore habitats (Figs 4 and 5). Specifically, the northern coastal reefs of the South China Sea, which experience high levels of human disturbance, exhibited lower microbial diversity and an enrichment of opportunistic microbial taxa in the skin microbiomes of reef fish. In contrast, offshore regions, characterized by lower turbidity, reduced terrestrial pollutants, and substantially lower anthropogenic impact [48], supported greater microbial diversity among fish species. Environmental differences between coastal and offshore habitats likely contribute to the observed shifts in microbial composition, while host habitat use mediates the selective colonization and assembly of skin microbiomes [16, 49]. These observations are consistent with our hypothesis that differences in temperature and turbidity, influenced by latitudinal gradients and terrestrial inputs, as well as anthropogenic disturbances, including nutrient enrichment and heavy metal pollution [50], drive the differences in coral reef fish skin microbial composition between coastal and offshore regions. Temperature and silicate concentration were identified as the primary environmental drivers of variation in the skin microbiomes of coral reef fish in the South China Sea. Temperature directly influences the diversity and composition of aquatic microbial communities, while also affecting host immune status and skin secretion profiles, thereby altering the microenvironment for microbial colonization [16, 51, 52]. Silicate availability influences the abundance of siliceous phytoplankton (e.g. diatoms), whereas chlorophyll-a, although not significant in the multivariate model, serves as a general indicator of primary production and microbial growth or proliferation in the water column. The proliferation of phytoplankton can modulate the structure of bacterial communities in the water column, ultimately altering the microbial exposure environment of fish [53]. These findings align with previous research demonstrating that both the magnitude and direction of environmental effects depend on fluctuations in these physiochemical parameters, as well as the physiological and behavioral adaptability of the host fish [45].

Consistent with these environmentally driven shifts in community structure, predicted functional profiles also differed between habitats, with coastal microbiomes showing enrichment of pathways related to bacterial chemotaxis and Vibrio-associated processes, whereas offshore communities exhibited relatively higher representation of diverse metabolic pathways, including amino acid metabolism and biosynthetic functions. These patterns suggest that environmental gradients may influence not only which taxa colonize fish skin but also the potential metabolic strategies of these microbial assemblages. Although these predicted functional differences provide insight into potential metabolic shifts between habitats, it is important to recognize that PICRUSt2-based inference from 16S rRNA data represents an estimation of genomic potential rather than direct evidence of functional expression [54].

The presence of host-microbiome evolutionary associations may be due to selective pressures that retain beneficial microbial taxa within host lineages, thereby maintaining advantageous symbiotic microbes through both ecological and evolutionary mechanisms [55]. Similar to other studies on marine fish [22], our study found that the skin microbiomes of reef fish exhibit a phylosymbiotic pattern to some extent, although the Mantel tests showed a weak phylogenetic signal associated with the skin microbiome of reef fish (*r* = 0.1599, *P* < .01). While phylosymbiosis reflects congruence between host phylogeny and microbial community composition, it does not by itself demonstrate strict host-microbe co-diversification. Our results further suggest that the strength of phylosymbiosis patterns varies across different taxa [56], with certain coral reef fish families, such as Holocentridae and Kyphosidae, showing relatively stronger phylogenetic signals. Such lineage-specific patterns, also supported by LIPA analyses, may reflect conserved ecological strategies within particular clades, such as the predominantly nocturnal behavior and strong association with structurally complex reef microhabitats in Holocentridae, which may reinforce host-specific filtering of skin-associated microbial communities. Consistent with this interpretation, our additional analyses demonstrated that key host traits, including body size, mobility, activity period, and diet, exhibited significant phylogenetic signal, indicating that important ecological characteristics are conserved across reef fish lineages. Accordingly, the observed association between microbiome dissimilarity and host phylogenetic distance likely reflects not only shared evolutionary history but also correlated ecological and life-history traits that shape microbial community assembly.

The phylosymbiotic pattern in animal microbiomes may arise from multiple factors, including phenotypic divergence among phylogenetically distant hosts [15], coevolution between specific bacteria and their hosts [57], vertical transmission of bacterial lineages across host generations [58], and host behaviors or life history traits such as dietary preferences [59]. Different reef fish species occupy distinct ecological niches within coral reef ecosystems and have evolved diverse feeding strategies, adaptive traits, and behavioral characteristics [60, 61]. The differences in these functional traits among fish partially reflect their distinct patterns of microhabitat utilization and food resource exploitation, which in turn indirectly influence the composition and dynamics of the skin microbiome in coral reef fish [16]. Mobility, which reflects the degree of dependence on coral reefs, may also play a pivotal role in modulating host–microbe interactions. Fish species with a higher reliance on reef habitats tend to establish persistent associations with coral microbial communities, resulting in skin microbiomes that are significantly influenced by coral-associated microbes [62]. The effect of diet on the structure of skin microbiomes of coral reef fish may stem from diet-induced differences in skin mucus composition, which drives changes in the structure of the skin microbiome [12]. Additionally, dietary preferences may alter patterns of habitat use, leading to variation in environmental microbial exposure, which in turn shapes the structure of skin microbiomes.

To our knowledge, this is the first study to investigate the diversity and phylosymbiosis pattern of skin microbiomes in coral reef fish on a large spatial scale, encompassing a wide range of species and taxa. Our findings highlight the critical roles of both environmental and host-specific factors in shaping the skin microbiomes of coral reef fish. These results further support the conclusion that environmental and host-specific factors are both primary drivers of the skin microbiomes in animals [12]. Unlike non-coral reef fish [53], coral reef fish are highly dependent on their complex and specialized habitats [23], which uniquely shape the structure of their microbiomes. We demonstrate a weak phylosymbiotic signal in the skin microbiomes of coral reef fish, with evolutionary relationships among host species partially mediating the assembly of microbial communities. In other words, environmental factors directly dominate the skin microbiomes of coral reef fish, whereas host-specific factors exert indirect effects by regulating host’s microenvironment and food resource utilization. This research provides novel and valuable insights into the factors shaping the diversity of fish skin microbiomes, emphasizing the complex interplay between environmental influences and host-specific factors in coral reef ecosystems.

## Conclusion

This study systematically analyzes the diversity patterns of skin microbiomes of coral reef fish in the South China Sea, and identifies significant differences in the composition, diversity, and functionality of fish skin microbiomes across different habitats (coastal vs. offshore). The formation of coral reef fish skin microbiomes is influenced by complex interactions between environmental and host-specific factors, which collectively shape the complexity and diversity of microbial communities. Specifically, temperature and silicate concentration were the primary environmental factors affecting the skin microbial community structure of coral reef fish in the South China Sea, while host-specific factors such as mobility and diet indirectly contributed to the skin microbial community structure by regulating host’s microenvironment and feeding behaviors. In the context of global warming and increasing human disturbance, it is crucial to consider the impact of these factors on the health of coral reef fish and the stability of coral reef ecosystems. The dominant role of environmental filtering in microbial community assembly underscores the importance of understanding habitat-specific drivers, while the influence of host-specific traits highlights the significance of host autoregulation and plasticity. This study provides critical insights into how fish skin microbiomes respond to environmental variation and enhances our understanding of the mechanisms underlying host–microbe interactions, which are essential for the conservation of coral reef fish and the maintenance of coral reef ecosystem stability amid increasing global change.

## Supplementary Material

Supplementary_material_ycag121

## Data Availability

All data supporting the findings of this study are provided in the Supplementary Information and Supplementary Datasets. Specifically, Supplementary Dataset 1 provides detailed host trait and sample information for the coral reef fish skin microbiomes; Supplementary Dataset 2 includes environmental data from various sampling sites; Supplementary Dataset 3 presents the Amplicon Sequence Variant (ASV) abundance table derived from *16S rRNA* gene sequences; Supplementary Dataset 4 contains the rarefied ASV table (15 142 reads per sample, n = 447) used for alpha and beta diversity analyses; Supplementary Dataset 5 contains the predicted metagenome abundance table at the KEGG Ortholog (KO) level for all samples; Supplementary Dataset 6 contains the predicted pathway abundance table with KEGG pathway descriptions for all samples; and Supplementary Dataset 7 provides a metadata table linking the raw sequencing file names and run accessions in GSA to the corresponding sample identifiers used in the manuscript. The raw *16S rRNA* gene sequencing data have been deposited in the Genome Sequence Archive (GSA) in National Genomics Data Center, China National Center for Bioinformation / Beijing Institute of Genomics, Chinese Academy of Sciences (GSA: CRA023888), and are publicly accessible at https://ngdc.cncb.ac.cn/gsa. The R scripts used for the main statistical analyses and figure generation are publicly available through Zenodo (DOI: 10.5281/zenodo.19504976).
