# Supplementary material for: Exploring phylosymbiosis in the skin microbiome of coral reef fish: synergistic effects of environmental and host-specific factors
Source: ISME Commun. 2026 May 7;6(1):ycag121. doi: 10.1093/ismeco/ycag121 (PMC13196597; doi:10.1093/ismeco/ycag121)
Supplement: Supplementary_material_ycag121 [file supplementary_material_ycag121.zip › Supplementary Information.docx]

**Supplementary Information**

**Exploring phylosymbiosis in the skin microbiome of coral reef fish: Synergistic effects of environmental and host-specific factors**

**Supplementary Table 1. Summary of sequence processing and filtering steps for 16S rRNA amplicon data**

| **Step** | **Description** | **Number of reads retained** | **% of total raw reads** |
| --- | --- | --- | --- |
| Raw reads | Total number of paired-end reads before any processing | 62,490,244 | 100% |
| Denoising | Adapter and primer trimming, chimera removal, quality filtering, and denoising | 16,410,614 | 26.26% |
| Taxonomic filtering | Removal of non-target sequences (e.g., mitochondria and chloroplasts) | 16,296,967 | 26.08% |
| Abundance filtering | Removal of low-abundance ASVs (<10 total reads) | 16,082,140 | 25.74% |
| Final ASV table (rarefied) | Rarefied to 15,142 reads per sample (n = 447 samples) | 6,768,474 | 10.83% |

**Supplementary Table 2. Classification of host traits and data sources.**

| **Traits** | **Classification** | **Data sources** |
| --- | --- | --- |
| Body size | 1. 0–7 cm 2. 7.1–15 cm 3. 15.1–30 cm 4. 30.1–50 cm 5. 50.1–80 cm 6. >80 cm | FishBase;  (Parravicini et al., 2021) |
| Mobility | 1. sedentary 2. mobile within a reef 3. mobile between reefs | FishBase;  (Parravicini et al., 2021) |
| Active period | 1. diurnal 2. both 3. nocturnal | FishBase;  (Parravicini et al., 2021; Siqueira et al., 2021) |
| Diet | 1. Herbivores, Microvores, Detritivores (HMD) 2. Corallivores 3. Piscivores 4. Sessile invertivores 5. Microinvertivores 6. Macroinvertivores 7. Crustacivores 8. Planktivores | FishBase;  (Parravicini et al., 2021; Parravicini et al., 2020) |

**Supplementary Table 3. Pairwise PERMANOVA comparisons among host trait categories for fish skin microbiome composition.**

| **Trait** | **Comparison** | **F** | **R^2^** | **P** | **P-adjusted** |
| --- | --- | --- | --- | --- | --- |
| Host size | 15.1-30 cm vs 50.1-80 cm | 3.57 | 0.0155 | 0.0010 | 0.0014 |
|  | 15.1-30 cm vs 30.1-50 cm | 1.73 | 0.0054 | 0.0140 | 0.0140 |
|  | 15.1-30 cm vs 7.1-15 cm | 6.02 | 0.0220 | 0.0010 | 0.0014 |
|  | 15.1-30 cm vs >80 cm | 3.02 | 0.0147 | 0.0010 | 0.0014 |
|  | 15.1-30 cm vs 0-7 cm | 3.26 | 0.0159 | 0.0010 | 0.0014 |
|  | 50.1-80 cm vs 30.1-50 cm | 2.63 | 0.0164 | 0.0020 | 0.0023 |
|  | 50.1-80 cm vs 7.1-15 cm | 5.12 | 0.0457 | 0.0010 | 0.0014 |
|  | 50.1-80 cm vs >80 cm | 3.29 | 0.0743 | 0.0010 | 0.0014 |
|  | 50.1-80 cm vs 0-7 cm | 4.18 | 0.0945 | 0.0010 | 0.0014 |
|  | 30.1-50 cm vs 7.1-15 cm | 5.33 | 0.0261 | 0.0010 | 0.0014 |
|  | 30.1-50 cm vs >80 cm | 3.19 | 0.0234 | 0.0010 | 0.0014 |
|  | 30.1-50 cm vs 0-7 cm | 3.28 | 0.0242 | 0.0010 | 0.0014 |
|  | 7.1-15 cm vs >80 cm | 2.38 | 0.0282 | 0.0010 | 0.0014 |
|  | 7.1-15 cm vs 0-7 cm | 1.72 | 0.0208 | 0.0080 | 0.0086 |
|  | >80 cm vs 0-7 cm | 2.64 | 0.1495 | 0.0020 | 0.0023 |
| Host activity | diurnal vs nocturnal | 5.53 | 0.0147 | 0.0010 | 0.0010 |
|  | diurnal vs both | 2.46 | 0.0072 | 0.0010 | 0.0010 |
|  | nocturnal vs both | 3.61 | 0.0198 | 0.0010 | 0.0010 |
| Host diet | Microinvertivores vs HMD | 3.13 | 0.0193 | 0.0010 | 0.0028 |
|  | Microinvertivores vs Crustacivores | 3.30 | 0.0196 | 0.0010 | 0.0028 |
|  | Microinvertivores vs Planktivores | 1.62 | 0.0163 | 0.0160 | 0.0213 |
|  | Microinvertivores vs Corallivores | 1.90 | 0.0285 | 0.0010 | 0.0028 |
|  | Microinvertivores vs Sessile invertivores | 1.80 | 0.0262 | 0.0030 | 0.0060 |
|  | Microinvertivores vs Piscivores | 2.15 | 0.0290 | 0.0020 | 0.0043 |
|  | Microinvertivores vs Macroinvertivores | 1.91 | 0.0313 | 0.0050 | 0.0082 |
|  | HMD vs Crustacivores | 3.51 | 0.0143 | 0.0010 | 0.0028 |
|  | HMD vs Planktivores | 2.36 | 0.0133 | 0.0010 | 0.0028 |
|  | HMD vs Corallivores | 1.81 | 0.0126 | 0.0070 | 0.0103 |
|  | HMD vs Sessile invertivores | 2.25 | 0.0154 | 0.0020 | 0.0043 |
|  | HMD vs Piscivores | 1.78 | 0.0118 | 0.0050 | 0.0082 |
|  | HMD vs Macroinvertivores | 1.19 | 0.0087 | 0.1940 | 0.1960 |
|  | Crustacivores vs Planktivores | 2.15 | 0.0118 | 0.0010 | 0.0028 |
|  | Crustacivores vs Corallivores | 2.81 | 0.0187 | 0.0010 | 0.0028 |
|  | Crustacivores vs Sessile invertivores | 2.56 | 0.0168 | 0.0010 | 0.0028 |
|  | Crustacivores vs Piscivores | 2.44 | 0.0155 | 0.0020 | 0.0043 |
|  | Crustacivores vs Macroinvertivores | 1.20 | 0.0084 | 0.1960 | 0.1960 |
|  | Planktivores vs Corallivores | 1.99 | 0.0239 | 0.0040 | 0.0075 |
|  | Planktivores vs Sessile invertivores | 2.18 | 0.0256 | 0.0010 | 0.0028 |
|  | Planktivores vs Piscivores | 1.47 | 0.0165 | 0.0620 | 0.0694 |
|  | Planktivores vs Macroinvertivores | 1.41 | 0.0184 | 0.0740 | 0.0797 |
|  | Corallivores vs Sessile invertivores | 1.71 | 0.0330 | 0.0120 | 0.0168 |
|  | Corallivores vs Piscivores | 1.73 | 0.0305 | 0.0070 | 0.0103 |
|  | Corallivores vs Macroinvertivores | 1.66 | 0.0380 | 0.0180 | 0.0219 |
|  | Sessile invertivores vs Piscivores | 2.38 | 0.0400 | 0.0010 | 0.0028 |
|  | Sessile invertivores vs Macroinvertivores | 1.57 | 0.0344 | 0.0190 | 0.0222 |
|  | Piscivores vs Macroinvertivores | 1.69 | 0.0334 | 0.0170 | 0.0216 |
| Host mobility | mobile within a reef vs sedentary | 7.05 | 0.0168 | 0.0010 | 0.0010 |
|  | mobile within a reef vs mobile between reefs | 4.03 | 0.0164 | 0.0010 | 0.0010 |
|  | sedentary vs mobile between reefs | 2.22 | 0.0094 | 0.0010 | 0.0010 |

**Supplementary Table 4. Phylogenetic signal of host traits (continuous variable).**

Phylogenetic signal of host body size was quantified using Blomberg's K and Pagel's λ based on 9999 permutations. Significant values indicate non-random phylogenetic conservatism.

| **Trait** | **Method** | **Estimate** | **P value** |
| --- | --- | --- | --- |
| Size | Blomberg's K | 0.535 | 1.0 × 10⁻⁴ |
| Size | Pagel's λ | 0.889 | 2.44 × 10⁻¹⁴ |

**Supplementary Table 5. Phylogenetic signal of host traits (categorical variables).**

Phylogenetic signal of host mobility, activity period, and diet guild was quantified using Fritz & Purvis' D statistic (9999 permutations).

D = 1 indicates random distribution across the phylogeny;

D = 0 corresponds to Brownian motion expectation;

D < 0 indicates stronger-than-Brownian phylogenetic clustering.

P values refer to deviation from random trait distribution.

| **Trait** | **Category** | **D** | **P (vs random)** |
| --- | --- | --- | --- |
| Mobility | Mobile between reefs | 0.193 | 0.0038 |
| Mobility | Mobile within a reef | −0.366 | <0.0001 |
| Mobility | Sedentary | −0.466 | <0.0001 |
| Activity | Both | −0.743 | <0.0001 |
| Activity | Diurnal | −0.722 | <0.0001 |
| Activity | Nocturnal | −0.747 | <0.0001 |
| Diet | Corallivores | −0.992 | <0.0001 |
| Diet | Crustacivores | −0.015 | <0.0001 |
| Diet | Herbivores, Microvores, Detritivores (HMD) | −0.676 | <0.0001 |
| Diet | Macroinvertivores | 0.186 | 0.0085 |
| Diet | Microinvertivores | 0.241 | 0.0004 |
| Diet | Piscivores | 0.074 | 0.0021 |
| Diet | Planktivores | 0.022 | <0.0001 |
| Diet | Sessile invertivores | −0.890 | <0.0001 |


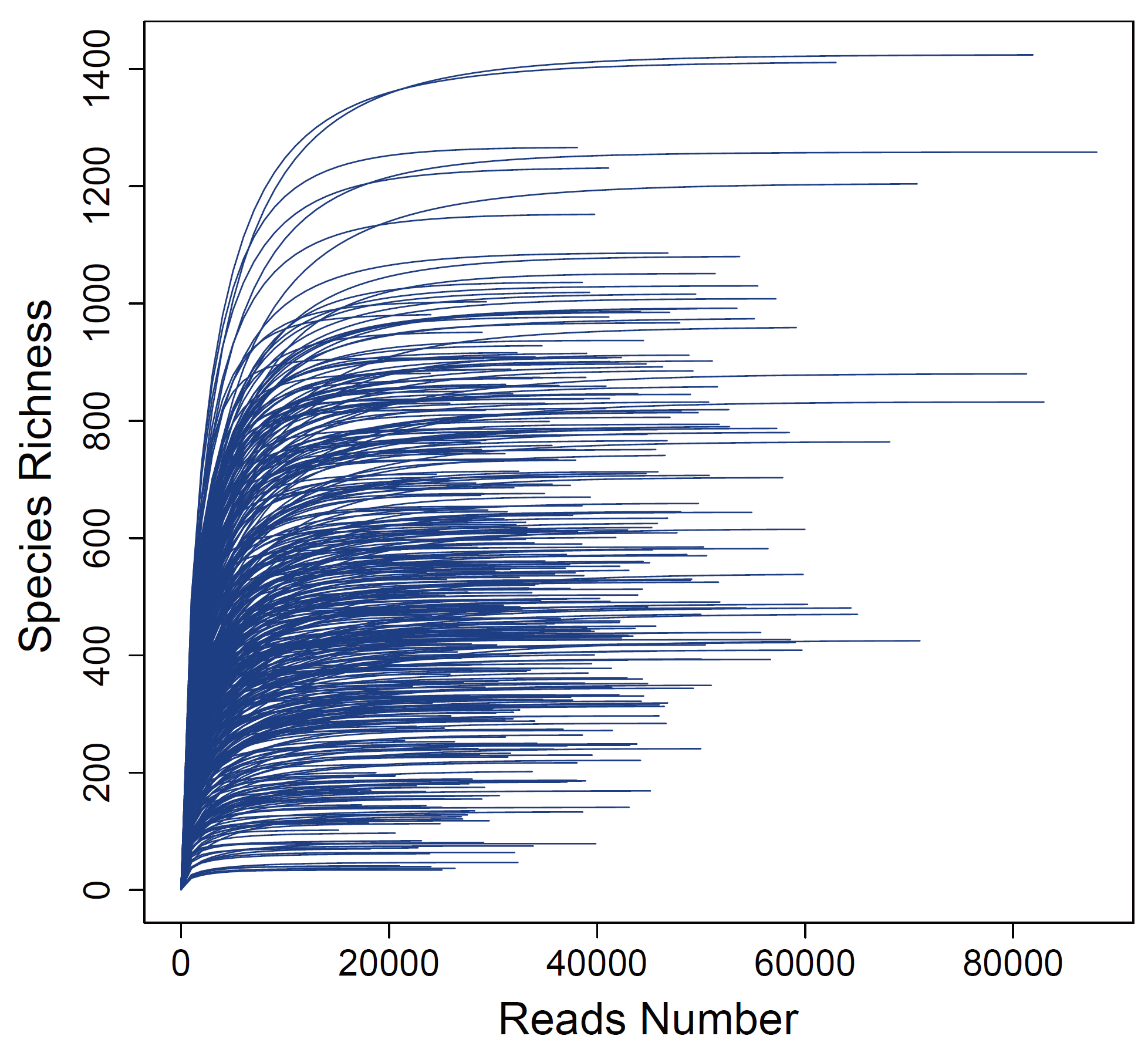


**Supplementary Figure 1. Rarefaction analysis of sampling depth for fish skin microbiome diversity.**


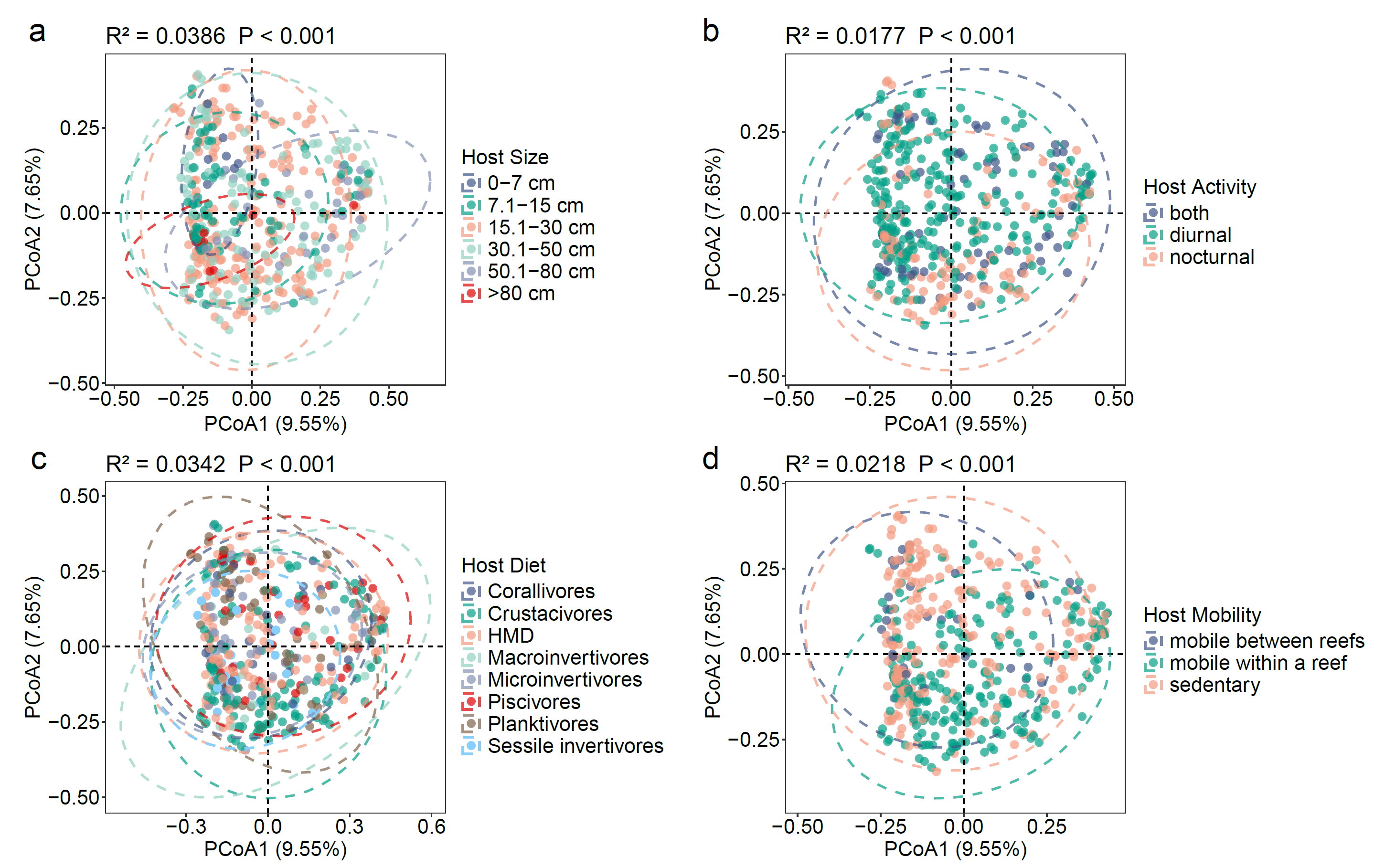


**Supplementary Figure 2. Principal coordinates analysis (PCoA) of Bray–Curtis dissimilarities in fish skin microbiome communities across host trait categories.** Points represent individual fish skin microbiome samples and are coloured by (a) host size, (b) host activity, (c) host diet, and (d) host mobility. Dashed ellipses indicate 95% confidence intervals for each group. Differences in community composition among trait categories were assessed using PERMANOVA (999 permutations), and the corresponding R^2^ and P values are shown in each panel. Detailed pairwise PERMANOVA comparisons are provided in Supplementary Table 3.


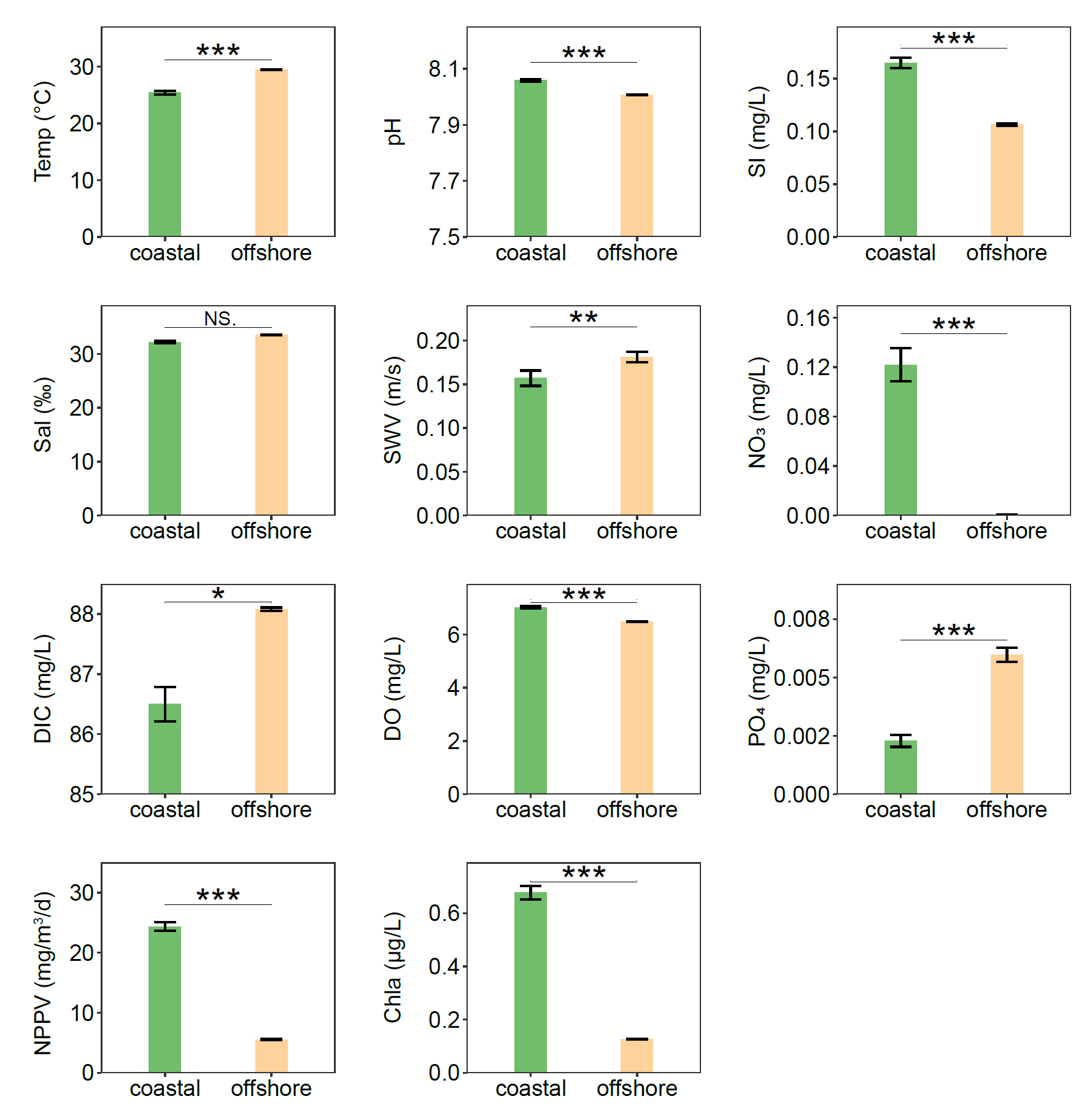


**Supplementary Figure 3. Comparison of environmental factors between coastal and offshore coral reef regions in the South China Sea.** Statistical significance levels are indicated by asterisks *(P* < 0.05: *; *P* < 0.01: **; *P* < 0.001: ***).


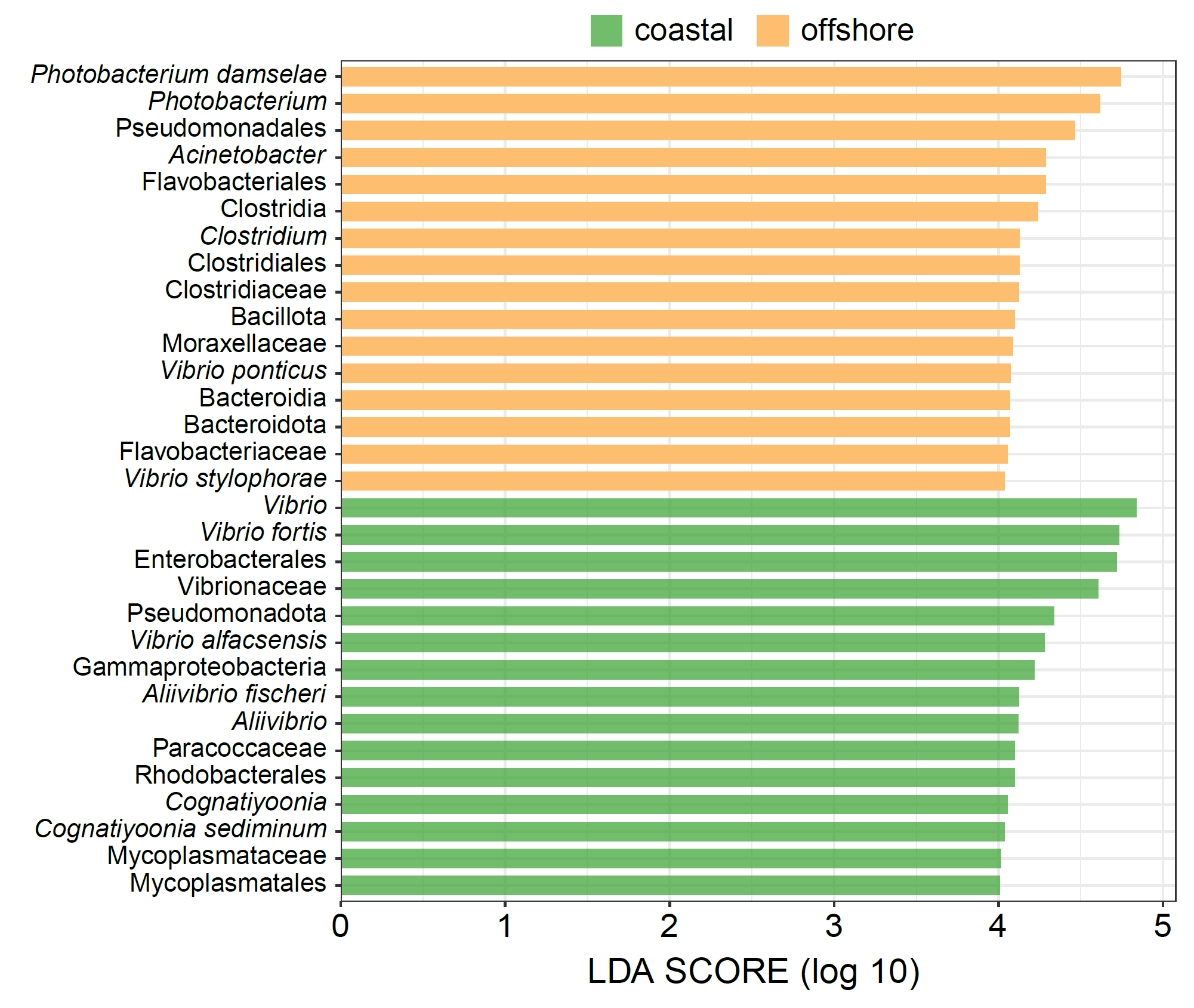


**Supplementary Figure 4. LEfSe analysis of coral reef fish skin microbiomes identifies habitat-specific microbial taxa.** Bar plot showing microbial taxa significantly enriched in coastal (green) and offshore (orange) habitats based on LEfSe analysis. Taxa with LDA scores > 4.0 are shown.


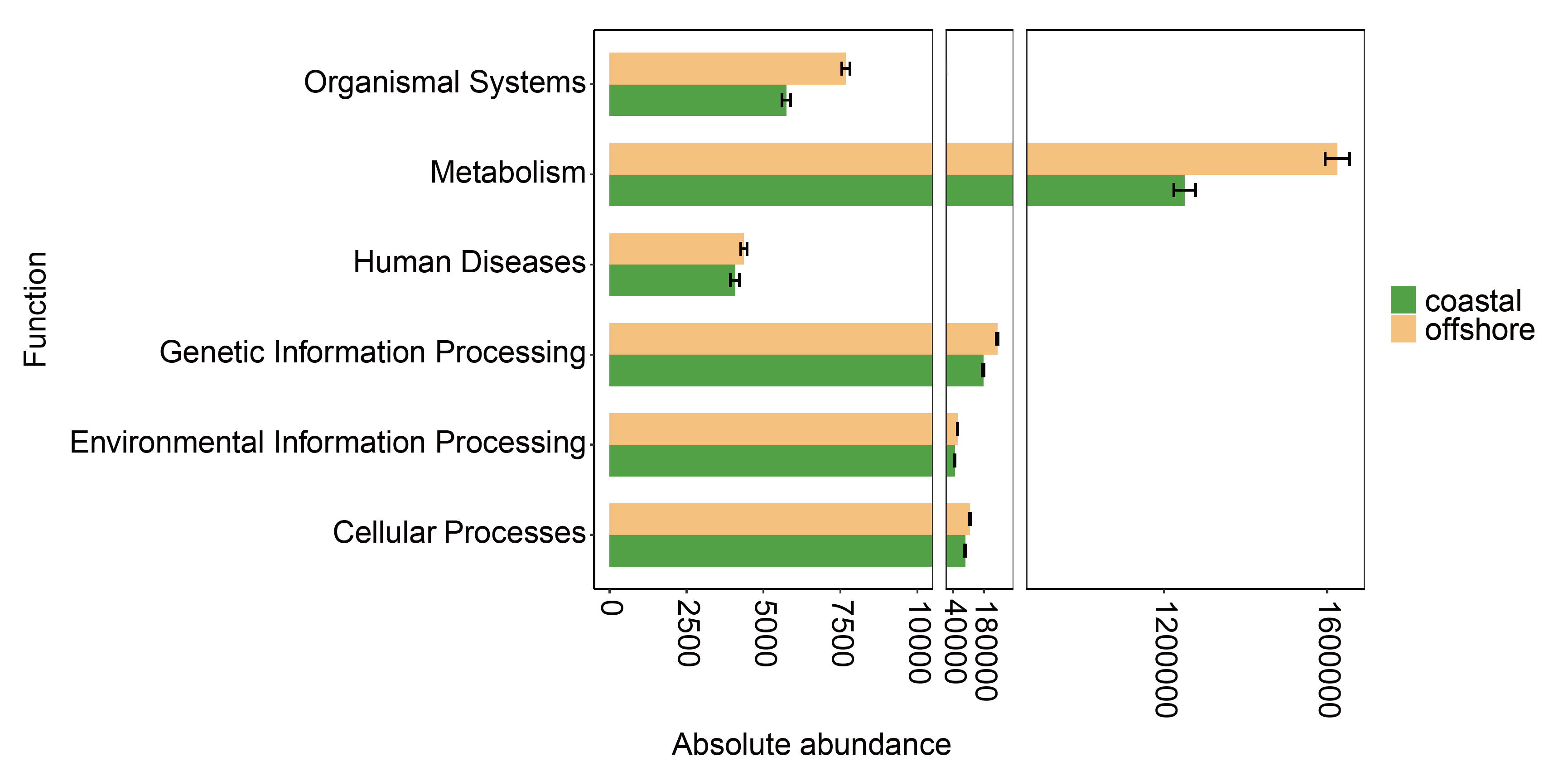


**Supplementary Figure 5. Distribution of KEGG Level 1 metabolic pathways in coral reef fish skin microbiomes from coastal and offshore habitats, based on absolute abundance.**
